# The Structural and Mechanical Properties of Al_2_O_3_–Ni Composites Obtained by Magnetic Field-Assisted Centrifugal Slip Casting

**DOI:** 10.3390/ma17163902

**Published:** 2024-08-06

**Authors:** Justyna Zygmuntowicz, Magdalena Kosiorek, Marcin Wachowski, Lucjan Śnieżek, Ireneusz Szachogłuchowicz, Paulina Piotrkiewicz, Waldemar Kaszuwara, Katarzyna Konopka

**Affiliations:** 1 Faculty of Materials Science and Engineering, Warsaw University of Technology, 141 Woloska St., 02-507 Warsaw, Poland; paulina.piotrkiewicz.dokt@pw.edu.pl (P.P.); waldemar.kaszuwara@pw.edu.pl (W.K.); katarzyna.konopka@pw.edu.pl (K.K.); 2 Institute of Power Engineering—National Research Institute, 8 Mory St., 01-330 Warsaw, Poland; magda.k@op.pl; 3 Institute of Heat Engineering, Faculty of Power and Aeronautical Engineering, Warsaw University of Technology, 21/25 Nowowiejska St., 00-665 Warsaw, Poland; 4 Faculty of Mechanical Engineering, Military University of Technology, 2 gen. S. Kaliskiego St., 00-908 Warsaw, Poland; marcin.wachowski@wat.edu.pl (M.W.); lucjan.sniezek@wat.edu.pl (L.Ś.); ireneusz.szachogluchowicz@wat.edu.pl (I.S.)

**Keywords:** Al_2_O_3_–Ni, composites, CSC, magnetic field, mechanical properties

## Abstract

This study investigates the influence of a magnetic field on the microstructure and properties of Al_2_O_3_–Ni composites fabricated via centrifugal slip casting at 1500 rpm. Al_2_O_3_ and Ni powders were combined with water and deflocculants, homogenized, and then cast into a porous plaster mold surrounded by Nd-Fe-B magnets. The resulting composites, sintered in a reducing atmosphere, exhibited a three-zone structure with varying Ni content due to the combined effects of the magnetic field and centrifugal force. SEM, EDX, and XRD analyses confirmed the distribution and composition of the phases. Hardness tests revealed the highest values at the outermost zone, with a gradual decrease toward the inner zones. Compression tests employing digital image correlation revealed high internal stresses and a significant improvement in compressive strength compared to non-magnetic field methods. This study confirms that magnetic field-assisted centrifugal slip casting significantly enhances the structural, hardness, and compressive strength properties of Al_2_O_3_–Ni composites, indicating promising potential for advanced applications.

## 1. Introduction

Functionally graded materials (FGM) are widely used in various fields due to their unique properties and versatility [1,2,3,4,5]. These materials, characterized by a gradual variation in composition and structure, are designed to meet specific performance requirements. One prominent application of FGMs is in structural components, such as turbine blades used in aerospace and power generation industries [6,7]. FGM also has applications in automotive applications, and interestingly, the applications for carbon nanotubes FGMs include 4D printing, soft robotics, electronics, and the growth of metamaterials [8,9,10,11,12,13,14,15]. The gradient composition of ceramics and metals in FGMs allows turbine blades to withstand high stresses from rotation at high angular speeds, endure extreme mechanical loads, and manage thermal gradients experienced during operation [8,9,10,11,12,13,14,15]. This combination ensures high resistance to cyclic thermal flux and provides enhanced thermal stability and mechanical performance, crucial for high-temperature applications [16,17,18,19].

FGMs are also used in the construction of pipes transporting toxic substances, where high chemical and corrosion resistance, internal hardness, and structural integrity are essential to prevent degradation and ensure safety under operational stresses [20,21,22,23,24,25]. Another significant application of FGMs is in aerospace, particularly for thermal shields in space vehicles [26,27]. These materials can effectively handle the thermal stresses encountered during re-entry and other high-temperature conditions in space. Additionally, FGMs find use in optoelectronics [28,29,30], where their ability to precisely control the gradient of properties is advantageous for devices that require specific optical and electronic characteristics [28,29,30].

Based on the latest literature, gradient materials can be produced using various methods, including self-supporting high-temperature synthesis [31], plasma spraying [7,32,33], impulse laser deposition [34,35], laminating [36], spray molding [37,38], electrolytic deposition [38,39,40], centrifugal slip casting [22], laser sintering [41,42], infiltration [43,44], and suspension coating [39,45].

One of the interesting methods of producing ceramic–metal gradient composites is Centrifugal Slip Casting (CSC) [22]. This technique utilizes the centrifugal force in combination with the classic slip casting method [22]. The use of porous gypsum molds allows the slurry to thicken due to capillary action. Several studies have focused on casting gradient composites from the Al_2_O_3_–Ni system using this method [46,47]. This process allows the production of three-zone materials, as examined in various works [48,49].

The influence of the solid phase content in the slurry on the properties of the obtained composite has been investigated, showing that higher solid phase content leads to wider individual zones [50]. Increased nickel concentration in a given zone reduces hardness but enhances fracture toughness due to the presence of plastic metal particles. The rotational speed used during centrifugal casting also affects the properties, with higher speeds resulting in a more distinct compositional gradient and improved mechanical properties [51]. The results show that the samples cast at lower rotational speed (800 rpm) exhibit lower relative density compared to those cast at higher rotational speed (1800 rpm).

Moreover, the properties of ceramic–metal composites produced using CSC also depend on the gypsum mold used. Research has shown that the absorption capacity of the mold influences the width of the zones containing metal particles [51]. Research shows that the higher the absorption capacity of the gypsum mold, the greater the width of the zone containing metal particles. Therefore, the width of the zones and properties such as open porosity, absorption capacity, and relative density of the composite depends on the gypsum mold used [52]. In another study [53], a system was used where a magnet was placed under the mold. The obtained results were compared with a sample cast without a magnetic field. In both cases, a change in the distribution of iron particles in the cross-section was obtained. In the case of the sample cast without a magnetic field, the particle distribution was influenced by gravity, which caused the concentration of iron particles in the lower part of the sample. However, a clear gradient appeared for the sample cast in a magnetic field. Additionally, along with the change in the distribution of iron particles in the sample, the arrangement of agglomerates of metal particles along the lines of the acting magnetic field was also observed.

All these studies on gradient composites are primarily cognitive work and require further supplementation. Therefore, in this work, a combination of slip casting and the use of a magnetic field was employed as a method to obtain gradient composites from the Al_2_O_3_–Ni system. This approach aims to expand the knowledge related to this topic.

The work investigated the influence of a magnetic field on the shaping of the microstructure and selected properties of Al_2_O_3_–Ni system composites obtained by centrifugal casting of slips at a centrifuge speed of 1500 rpm. Moreover, the study employed a system different from that previously used, enabling centrifugal casting of slip masses using a magnetic field.

## 2. Materials and Methods

Al_2_O_3_ powder (TM-DAR) and nickel powder (Alfa Aesar) were used in this work. The basic properties of the powders used in the production process are summarized in Table 1.

**Figure 1:**
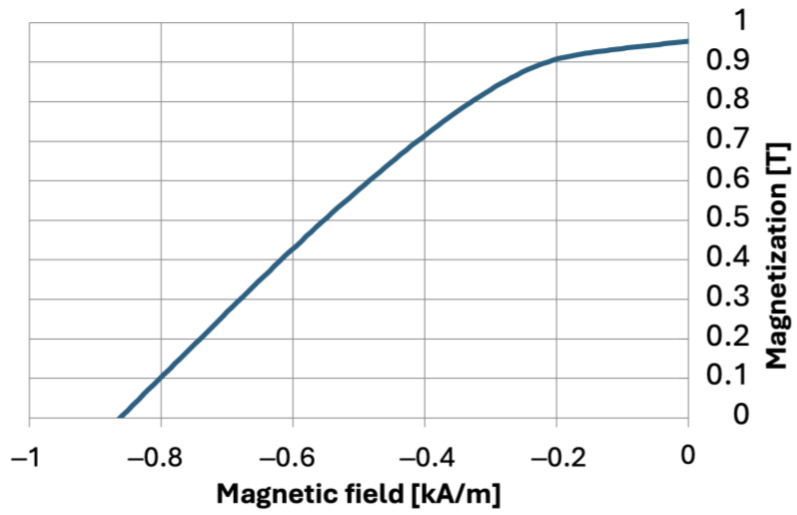
Demagnetization curve for the Nd-Fe-B magnets used in the experiment.

**Figure 2:**
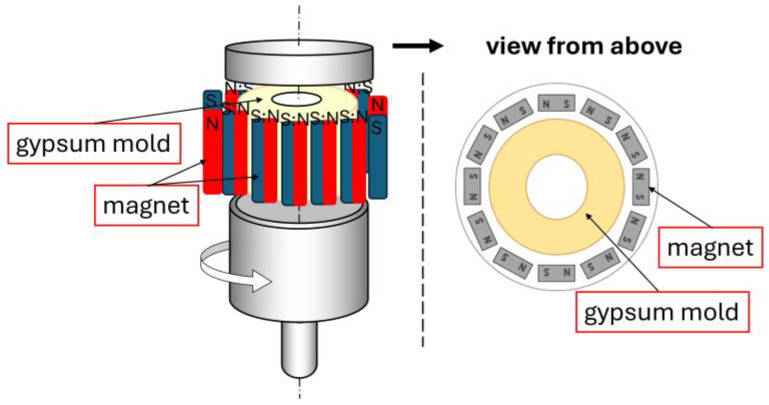
Scheme of the centrifugal casting system with a vertical axis of rotation in a magnetic field.

The composite production process involved multiple stages. Initially, the appropriate amounts of aluminum oxide and nickel powders were weighed and combined with deionized water and deflocculants. Distilled water was chosen as the solvent for economic and ecological reasons. Diammonium hydrogen citrate (DAC) was used as a fluidizing agent at 0.3% by weight, and citric acid (CA) in an amount of 0.1% by weight relative to the total powder mass, based on previous research findings [54,55]. The prepared suspension was homogenized using a THINKY ARE 250 high-speed mixer (LPP Equipment AG, Uster, Switzerland) in a multi-stage process, which included alternating mixing and deaeration: mixing at 2100 rpm for 8 min, deaeration at 1000 rpm for 2 min, stirring at 1500 rpm for 5 min, deaeration at 1000 rpm for 2 min, stirring at 500 rpm for 2 min, deaeration at 1000 rpm for 2 min. Following homogenization, the prepared suspension was poured into a porous plaster mold surrounded by neodymium (Nd-Fe-B) magnets. The experiment used commercial Nd-Fe-B magnets with the demagnetization curve shown in Figure 1 and the following properties: coercivity of 862 kA/m, remanence of 0.953 T, energy (BH) max of 140 kJ/m^3^. The magnetic field measured on the magnet surface was 0.25 T. The casting setup is illustrated in Figure 2. The slurry was subjected to rotation at a speed of 1500 rpm for 90 min using a vertical axis rotation system. The porous plaster mold containing the suspension was placed in a sleeve and tightly sealed. The arrangement of magnets supported the centrifugal force, enhancing the formation of a gradient in the composite.

Following casting and thorough drying, the composites were sintered in a reducing atmosphere (H_2_/N_2_). The parameters of the sintering process were determined experimentally based on previous results from thermogravimetric and dilatometric tests [46,47]. The deliberate choice was made to prevent the formation of oxides or spinels in the produced composites, which could occur if sintered in an oxidizing atmosphere such as air [56,57]. The sintering process proceeded in several stages. Initially, the furnace was heated at a rate of 5 °C/min to reach 120 °C. Subsequently, the temperature was increased at 1 °C/min until reaching 750 °C. Then, the heating rate was increased to 2 °C/min to 1400 °C, and the samples were maintained for 2 h. Finally, the samples were cooled to room temperature.

The magnetic properties of nickel powder were analyzed using a vibrating sample magnetometer (VSM), which operates based on Faraday’s law of electromagnetic induction. According to this law, when a closed circuit is placed in an alternating magnetic field, an electromotive force is induced that is proportional to the rate of change of the magnetic flux passing through the circuit’s surface [58,59]. In a VSM, the sample is positioned between magnetic coils and undergoes harmonic motion perpendicular to the applied magnetic field direction. This movement generates a varying magnetic flux, causing an electromotive force to be induced in the measuring coil, in accordance with Faraday’s law. This induced voltage is directly proportional to the magnetization of the sample being tested [57]. The values of coercivity, remanence, and magnetization saturation are determined on the basis of the magnetic hysteresis loops recorded in the study [57]. The nickel powder used for forming gradient-reinforced composites underwent these measurements using a LAKESHORE Vibra Cell 730931 vibration magnetometer (Newtown, CT, USA).

The Archimedes method was used to determine the selected physical properties of the obtained composites. The Archimedes method is a robust and practical approach to determining the relative density of composites, which is particularly useful for materials with irregular shapes. This method relying on the fundamental principles of buoyancy allows for accurate and straightforward measurement of density, which is essential in various scientific and engineering applications. In the experiment, the first stage of the study involved preparing composite samples. To do this, the samples were cut into 1 cm high fragments. Each fragment was then weighed in the air before being boiled in distilled water for 1 h to ensure water penetration into the material. After boiling, the samples were weighed in water at room temperature, dried, and then weighed again in air. These measurements were conducted to determine the relative density, open porosity, and water absorption parameters.

The morphology of the powders used in this study and the microstructure of the composites obtained after the sintering process were examined using a JSM-6610 scanning electron microscope (SEM), JEOL USA, INC., 11 Dearborn Road, Peabody, MA, USA. Following the sintering process, the samples for observation were cut appropriately using a saw with a diamond disc. The samples were cut in a plane transverse to the rotation axis. Then, the obtained sample fragments were incorporated into the resin for further preparation. Subsequently, the samples underwent grinding using an automatic grinder and polisher (Saphir 550). The grinding process involved sequential use of discs with different gradations: PLATINUM 0 disc (Buehler, Lake Bluff, IL 41 Waukegan Road Lake Bluff) (80–100 grit), PLATINUM 1 disc (Buehler, Lake Bluff, IL 41 Waukegan Road Lake Bluff) (120–180 grit), PLATINUM 2 disc (220–320 grit), PLATINUM 3 disc (Buehler, Lake Bluff, IL 41 Waukegan Road Lake Bluff) (600 grit), PLATINUM 4 disc (Buehler, Lake Bluff, IL 41 Waukegan Road Lake Bluff) (1200 grit).

The EDX analysis was performed to determine the chemical composition of the obtained samples.

XRD tests on both raw and sintered composites were performed using a Rigaku Miniflex II X-ray diffractometer (Rigaku, Osaka, Japan) with a CuKα anode. The measurements were carried out at a voltage of 30 kV and an intensity of 15 mA with an angular range of the θ (20°–100°), with a rotation of 0.05° and a counting time of 1 s.

Microhardness testing involved making impressions at regular intervals across the entire cross-section of the composite in order to obtain the microhardness distribution. The HVS-30T hardness tester from HUATEC Group Corporation (HUATEC GROUP CORPORATION, Beijing, China) was used, applying a pressure of 9.8 N for 10 s.

To determine the strength properties of hollow cylinder samples, a monotonic compression test was performed along with simultaneous registration of destruction processes using a digital image correlation system for measuring strain maps. The test was conducted using an Instron 8802 hydraulic pulsator (Instron, Norwood, MA, USA) equipped with specialized software for continuous recording of load as a function of the displacement of the plate compressing the sample. The cameras could record images at a frequency exceeding 100,000 Hz. The test yielded graphs showing the relationship between loading force and displacement of the compression plate during the monotonic load test.

## 3. Results and Discussion

### 3.1. Characteristics of Starting Materials

**Figure 3:**
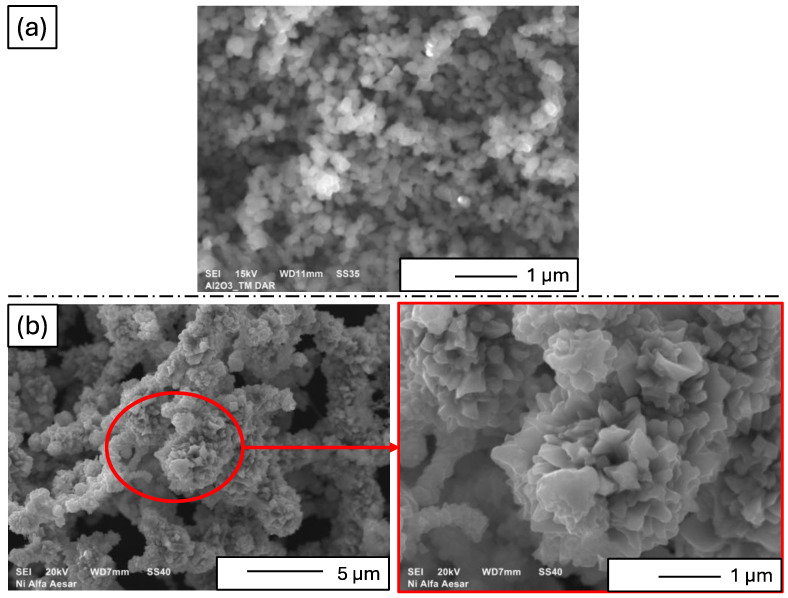
Examples of SEM micrographs showing the morphology of the starting powders: (**a**) aluminum oxide and (**b**) nickel.

The morphology of the starting powders is presented in Figure 3. SEM micrograph analysis indicated that both the Al_2_O_3_ and nickel powders tended to form agglomerates in the raw state. The grain sizes estimated on the basis of SEM micrographs were consistent with those provided by individual manufacturers. The Al_2_O_3_ exhibited spherical particles with a grain size of 100 nm ± 20 nm (Figure 3a). The SEM micrograph labeled Figure 3b presents the surface morphology of nickel powder. The nickel particles might be irregularly shaped. The image gives a general view of the particle arrangement and surface characteristics. The magnification of Figure 3b zooms in on a specific area of the nickel powder to reveal finer details that are not visible in the main micrograph. The magnified part shows a detailed view of the surface texture of the nickel particles. The observation reveal that nickel powder showed irregularly shaped particles with sharp edges and numerous protrusions, averaging 45 µm ± 20 µm in size (Figure 3b).

**Figure 4:**
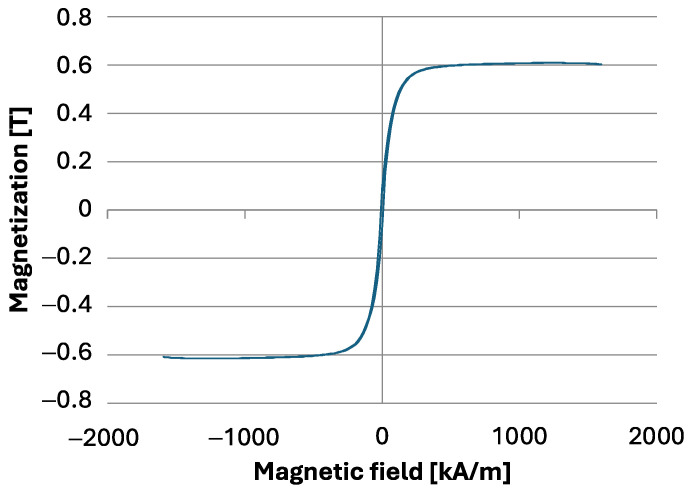
Hysteresis loop graph for nickel powder.

Figure 4 shows magnetic coercivity diagrams for nickel powder obtained using a vibrating magnetometer. Magnetic coercivity is defined as the external magnetic field required to reduce the residual magnetization to zero after demagnetizing the material from a saturated state [60,61]. For the tested powder, the coercivity was measured at Hc = 8.45 kA/m. The graph indicates a narrow hysteresis loop characteristic of the tested powder. The saturation magnetization value was Ms = 54.765 em/g (0.612 T), and the remanence was Br = 7.1092 emu/g. Based on the analysis, it can be concluded that nickel powder exhibits ferromagnetic properties and is responsive to external magnetic fields. This property makes it suitable as a reinforcing phase in centrifugally cast gradient composites, where the application of a magnetic field supports the formation of gradients. 

### 3.2. Characteristics of Composites—Microstructure

**Figure 5:**
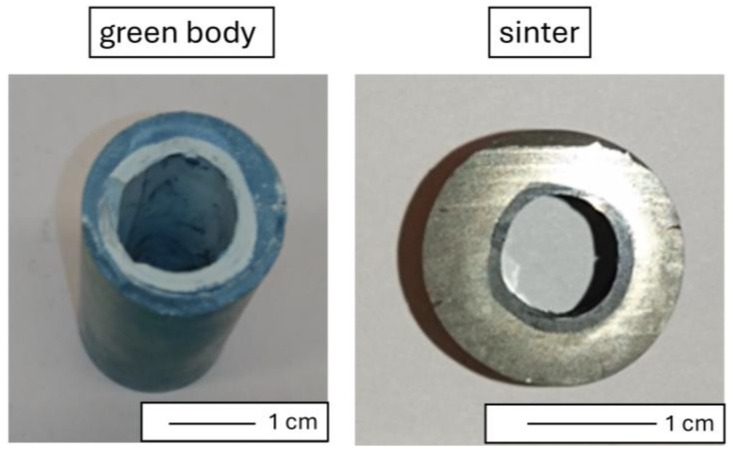
Macroscopic image of the sintered sample.

The method employed facilitated the production of a sleeve-shaped composite characterized by an apparent density of 4.30 ± 0.12 g/cm^3^. The relative density was 96.92 ± 3.12%. Open porosity was determined to be 0.66 ± 0.12%, with a water absorption rate of 1.83 ± 0.24%. Figure 5 shows the representative sintered sample used in the research. The obtained sample has dimensions: 35.7 mm of length, 15.9 mm of outer diameter, and 5.8 mm of inner diameter.

**Figure 6:**
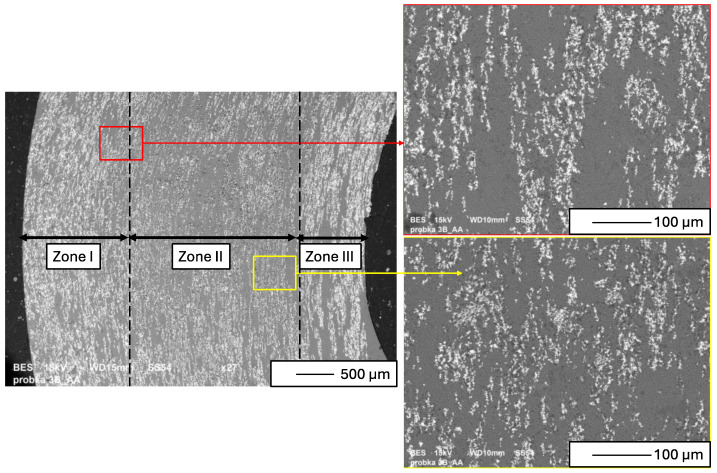
Cross-section of an Al_2_O_3_–Ni sample.

Figure 6 illustrates the microstructure of the cross-section of the produced Al_2_O_3_–Ni composite. In the SEM micrograph, bright areas correspond to the metallic phase, while grey areas represent the aluminum oxide matrix. The cross-section analysis revealed a non-uniform distribution of the metallic phase, extending from the outer to the inner edge of the composite with varying concentrations. The results indicate a three-zone structure within the Al_2_O_3_–Ni composite, distinguished by different nickel particle contents. It was assumed that Zone I is the outermost area covering 1320 µm. Zone II is an area of 2482 µm located inside the composite. The area inside the sleeve covering 526 µm was assumed to be zone III. The system for forming samples with a magnetic field used in the experiment allowed the creation of a composite that was characterized by the highest content of the metallic phase in Zone I and Zone III. Previous studies have shown that zone III contained mainly aluminum oxide [48,49,50,51]. Most likely, the use of a rotational speed of 1500 rpm made it impossible to obtain a zone characterized by the absence of a metallic phase, which could be observed in earlier works using a higher rotational speed to form samples [51,62,63]. Based on the micrographs obtained (Figure 6), the orientation of nickel particles in relation to the applied magnetic field was observed. It was found that the particles aligned along magnetic field lines and centrifuge rotation direction. These observations seem to confirm our working hypothesis that the centrifugal casting system used in the experiment with a magnetic field generated by a set of single-neodymium magnets allows for obtaining a gradient structure. Nevertheless, it is important to note that the lower rotational speed used (1500 rpm) in this study precluded the formation of a zone without metal particles. Therefore, preliminary tests demonstrated the suitability of the system used for creating gradient composites from the Al_2_O_3_–Ni system.

The distribution of metal particles in the obtained sample is not homogeneous due to several factors inherent in the manufacturing processes and the physical properties of the materials involved. A few reasons contribute to the non-uniform distribution of metal particles, such as centrifugal force, magnetic field application, viscosity and slurry properties, rotational speed, and porosity of the mold. The first factor is the fact that during the CSC process, the application of centrifugal force causes the denser nickel particles to move toward the outer regions of the composite. This movement is driven by the difference in density between the ceramic (Al_2_O_3_) and the metal (Ni) particles, with nickel being significantly denser (8.9 g/cm³) compared to alumina (3.95 g/cm³). As a result, nickel particles tend to concentrate at the periphery, creating a gradient distribution. In addition to centrifugal force, the use of a magnetic field further influences the distribution of nickel particles. Nickel is ferromagnetic and, when subjected to a magnetic field, the particles align along the magnetic field lines. This alignment, combined with centrifugal force, enhances the formation of a gradient structure, as observed with the chains of nickel particles aligning along the circumference of the composite. The properties of the slurry, including its viscosity and the solid phase content, affect the distribution of particles. A higher solid phase content in the slurry leads to a wider distribution of individual zones, as more particles are available to be distributed by centrifugal force and magnetic field influences. Additionally, the fluidizing agents and deflocculants used in the slurry preparation impact how well the particles are dispersed before casting. The rotational speed during the casting process significantly impacts the particle distribution. Higher rotational speeds enhance the centrifugal force, promoting a more pronounced gradient. Conversely, lower speeds may not sufficiently drive the nickel particles outward, resulting in a less distinct gradient. The study showed that samples cast at 1500 rpm had different structural characteristics compared to those cast at higher speeds like 1800 rpm. The properties of the porous gypsum mold also play a role. Molds with higher absorption capacities lead to greater widths of zones containing metal particles. The capillary action within the porous mold helps in thickening the slurry, which interacts with the centrifugal and magnetic forces to influence the final distribution of particles. These factors collectively lead to a non-homogeneous distribution of metal particles in FGMs, which is a deliberate and desirable feature in many applications to achieve specific mechanical and thermal properties tailored to operational demands. 

**Figure 7:**
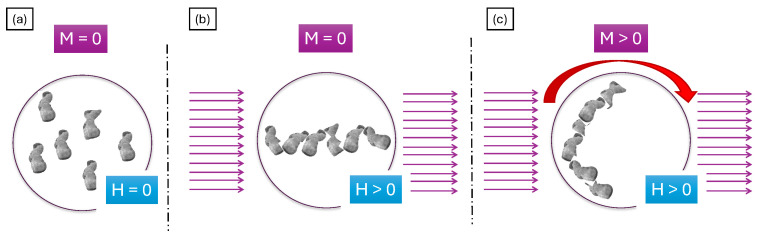
Mechanism of formation of the microstructure of metallic chains in Al_2_O_3_–Ni composites during the centrifugal casting process using a magnetic field. (**a**) M = 0, H = 0; (**b**) M = 0, H > 0; (**c**) M > 0, H > 0.

The microstructure of metal chains in the cross-section conforms to the model described in previous studies [64]. Figure 7 illustrates a diagram of the model describing the formation of a microstructure during centrifugal casting using a magnetic field. In the initial phase of the process, the centrifuge was turned off (rpm = 0, M = 0). In the absence of a magnetic field (H = 0), ferromagnetic nickel particles are separated and uniformly distributed in the suspension (Figure 7a). Upon activation of the magnetic field (H > 0), the ferromagnetic nickel powder becomes magnetized. Each nickel particle acts as a magnet in response to the magnetic field, leading them to attract and connect with their neighboring particles in chains (Figure 7b). As the centrifuge starts to rotate (M > 0), centrifugal force drives the chained particles toward the outer surface of the mold. The nickel particle chains position themselves to minimize the distance between their center of gravity and the mold wall, as depicted in Figure 7c. Eventually, chains of ferromagnetic particles (nickel particles) align along the circumference of the sample. The model presented in Figure 7 explains the process by which the composite microstructure shown in Figure 6 was achieved. 

### 3.3. Characteristics of Composites—Chemical and Phase Composition

**Figure 8:**
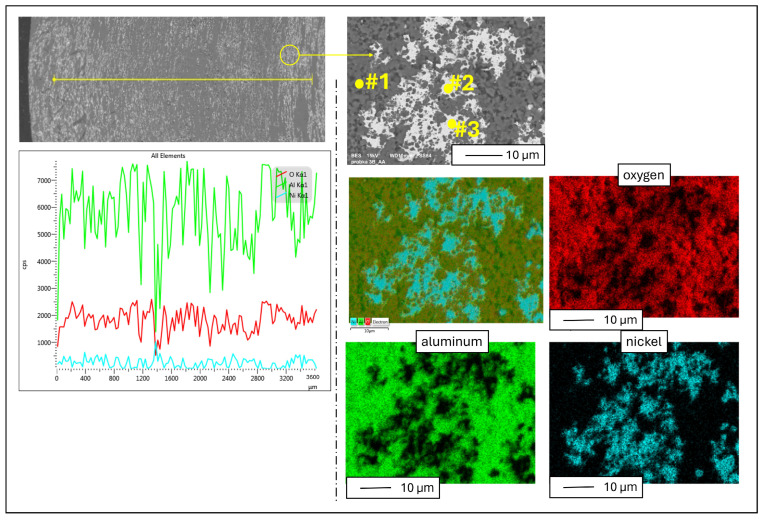
Analysis of the chemical composition of the Al_2_O_3_–Ni composite.

Subsequently, the EDX analysis was performed to determine the chemical composition, with results presented in Figure 8 and Table 2. On the left, Figure 8 shows a linear analysis of the chemical composition carried out on a cross-section of the samples. On the right, Figure 8 shows an enlargement of a selected area from the cross-section of the Al_2_O_3_–Ni composite, along with a detailed analysis of the chemical composition, including mapping and chemical composition from randomly selected points on the cross-section of the sample. The analysis revealed that the sample primarily consists of aluminum, oxygen, and nickel. Point EDX analysis confirmed that the bright areas in the microstructure correspond to the metallic phase. Specifically, at point #2, the analysis yielded the following composition: aluminum 1.81 wt.%, oxygen 2.09 wt.%, and nickel 96.09 wt.%, indicative of the metallic phase content. Similar compositions were found at point #3 (marked in Figure 8), to which the following values were assigned: aluminum 1.00 wt.%, oxygen 1.51 wt.%, nickel 97.94 wt.% responsible for the presence of the metallic phase. Conversely, at point #1 (marked in Figure 8), the composition revealed aluminum at 33.14 wt.% and oxygen at 66.86 wt.%, consistent with the composite matrix composed of aluminum oxide.

**Figure 9:**
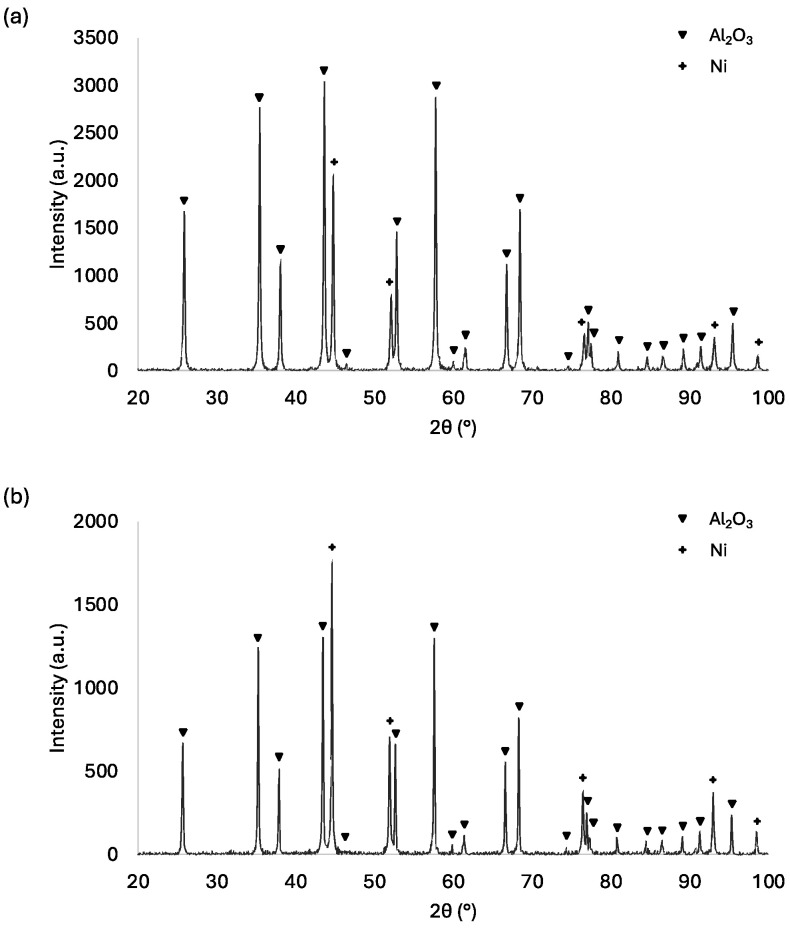
XRD analysis of the Al_2_O_3_–Ni sample in the raw state (**a**) and after the sintering process (**b**).

The XRD analysis results obtained are presented in Figure 9. XRD analysis of the Al_2_O_3_–Ni sample, both in its raw state and after the sintering process, revealed the presence of two phases in the material: Al_2_O_3_ (PDF #98-000-0174) and Ni (PDF #04-016-4261).

Detailed analysis of the diffractograms acquired as a result of the measurement enabled the identification of five peaks characterizing the metallic phase.

For the raw sample measurements, reflections originating from the families of crystallographic planes (111), (200), (220), (311), and (222) were observed at 2ϴ angles of 44.60°, 51.95°, 76.46°, 92.99°, and 98.49°, respectively. In the sintered sample, similar reflections from these crystallographic planes were observed at 2ϴ angles of 44.76°, 52.10°, 76.60°, 93.12° and 98.60°.

### 3.4. Characteristics of Composites—Hardness

**Figure 10:**
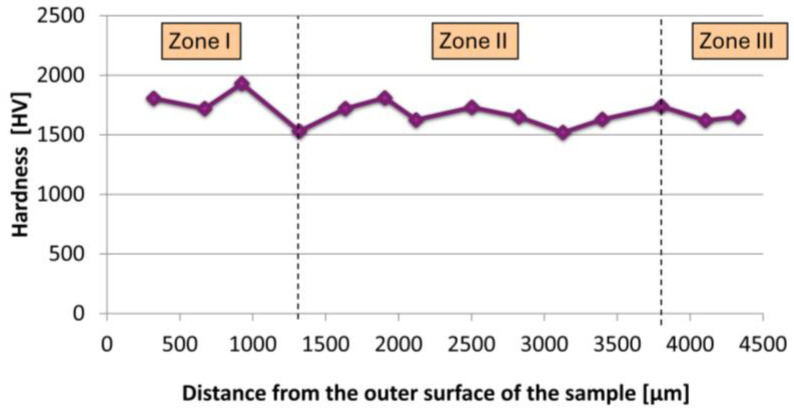
Hardness measurement graph on a cross-section for the Al_2_O_3_–Ni composite.

Then, the focus shifted to conducting a hardness test on the cross-section of the composite. The results obtained are presented in Figure 10. The hardness test revealed significant variations across different zones of the composite structure. The values of measurements are presented in Table 3. In zone I, which corresponds to the outermost edge of the sample, the composite exhibited the highest average hardness of 1746.25 ± 145.49 HV (17.83 ± 1.42 GPa). Moving inward to zone II, the average hardness slightly decreased to 1669.29 ± 86.37 HV (16.37 ± 0.84 GPa). Here, the distribution of nickel particles was still evident but less dense compared to zone I. In zone III, located closest to the inner part of the composite, the hardness further decreased to 1635 ± 15 HV (16.13 ± 0.05 GPa). It is evident that moving away from the outer edge of the sample, the hardness of the composite decreased, which is likely attributed to the formation of nickel particle chains during the casting process.

### 3.5. Characteristics of Composites—Compression Test Results

**Figure 11:**
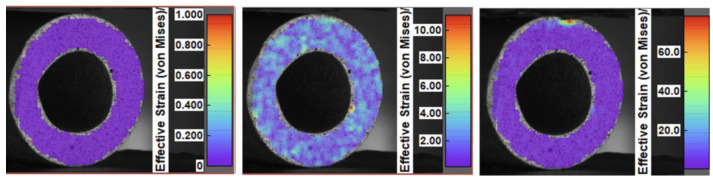
Strain distribution for the Al_2_O_3_–Ni sample using the digital image correlation method.

Figure 11 shows the strain distribution for the Al_2_O_3_–Ni sample using the digital image correlation method. The tested composite exhibited excellent resistance to deformation. No structural changes or displacements were observed throughout the entire study. The sample failure was highly dynamic, leading to its fragmentation. This behavior indicates the presence of significant internal stresses within the sample, likely induced by the manufacturing process used.

**Figure 12:**
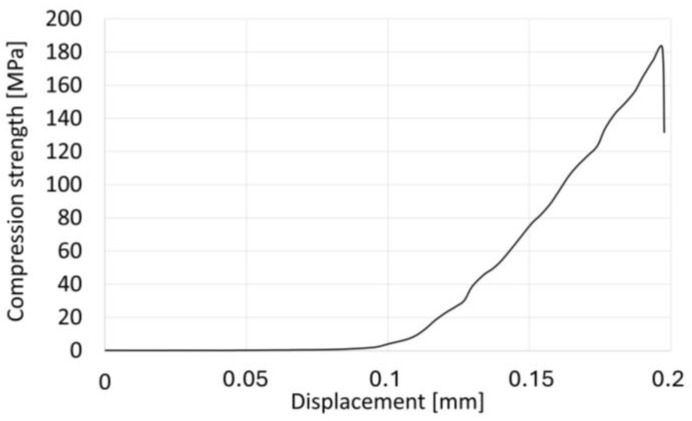
Diagram of average for multiple samples deformation as a result of monotonic compression for the Al_2_O_3_–Ni sample.

Figure 12 shows the results of the monotonic compression test for the tested composite. The graph shows the dependence of stress on the displacement of the piston in the testing machine. Upon investigating the course of the curve, it was observed that initially, there was a slight slope in the load, followed by a more linear phase. Toward the end, there was a sharp drop in the load, indicative of dynamic brittle fracture initiation in the sample, as confirmed by compression tests using digital image correlation (Figure 11). The observed linear increase in load for the tested material proves the absence of structural artifacts in the composites. The compressive strength values obtained were found to be higher than those reported in previous research [63] on ceramic–metal composites. Specifically, for the Al_2_O_3_–Ni composites studied here, the compressive strength was determined to be 182.53 MPa.

Furthermore, it was also found that the method of producing composites using a magnetic field allows for obtaining composites with higher compressive strength compared to the centrifugal slip casting method without a magnetic field, as reported for composites in the ceramic–metal system [63]. Previous studies have shown that Al_2_O_3_–Ni composites produced via centrifugal slip casting with 50% vol. of the solid phase and 10% vol. of the metallic phase achieved a compressive strength of 42.45 MPa [63]. Therefore, the introduction of a magnetic field significantly enhances the orientation of the metallic phase within the composite structure, resulting in a substantial increase in compressive strength.

The study demonstrates that the Al_2_O_3_–Ni composite exhibits a gradient in hardness from the outer edge to the inner part, excellent resistance to deformation, and significantly higher compressive strength compared to composites produced by centrifugal slip casting. The application of a magnetic field during the production process plays a crucial role in enhancing the mechanical properties of the composite.

## 4. Conclusions

The study investigated the influence of a magnetic field on the formation and properties of Al_2_O_3_–Ni gradient composites produced by centrifugal slip casting. These composites, crucial for applications under high-stress and high-temperature conditions, are produced using a combination of ceramics and metals. The experimental approach involved mixing Al_2_O_3_ and Ni powders with water and deflocculants, followed by casting them into a mold under the influence of both a magnetic field and centrifugal force. The process aimed to achieve a gradient distribution of Ni particles within the composite.

Key findings from the study include the following:

- The centrifugal casting with a magnetic field resulted in a structured composite with three distinct zones characterized by varying concentrations of nickel particles;
- The highest hardness was observed in the outermost zone, while the innermost zone exhibited lower values;
- The magnetic field induced the alignment of nickel particles, forming chains along the field lines and enhancing the gradient structure;
- The produced composite demonstrated excellent compressive strength (182.53 MPa), significantly exceeding counterparts produced without magnetic field assistance;
- The research highlights the effectiveness of integrating a magnetic field into the centrifugal slip casting process to produce gradient composites with improved structural and mechanical properties, suggesting the potential for enhanced performance in various industrial applications.

These findings contribute to advancing the understanding and application of magnetic-field-assisted manufacturing techniques for enhancing the performance of ceramic–metal gradient composites. The demonstrated improvements in mechanical strength and structural integrity highlight the potential of these materials for various industrial applications requiring robust and durable materials.

## Figures and Tables

**Table 1:** Characteristics of starting powders based on manufacturer’s data.

Properties	Al_2_O_3_	Ni
Grain size	100 nm ± 20 nm	~325 mesh (ok. 44 µm)
Purity	>99.9%	99.8%
Density	3.95 g/cm^3^	8.9 g/cm^3^
Melting point	2072 °C	1455 °C
Manufacturer	Tiamei Japan, Tokyo, Japan	Alfa Aesar, Shanghai, China
Trade name	TM-DAR	-

**Table 2:** The chemical composition of the samples (Figure 8) was determined by EDX analysis.

	Chemical Composition					
Point [Figure 8]	Aluminium		Oxygen		Nickel	
	Weight %	Atomic %	Weight %	Atomic %	Weight %	Atomic %
#1	33.14 ± 0.19	45.53 ± 0.09	66.86 ± 0.19	54.47 ± 0.02	-	-
#2	1.81 ± 0.06	3.67 ± 0.03	2.09 ± 0.07	7.12 ± 0.12	96.09 ± 0.10	89.21 ± 0.21
#3	1.00 ± 0.06	2.06 ± 0.09	1.51 ± 0.07	5.27 ± 0.13	97.49 ± 0.09	92.66 ± 0.04

**Table 3:** The values of hardness measurements in different zones.

Zone	Average Hardness (HV)	Standard Deviation (HV)	Average Hardness (GPa)	Standard Deviation (GPa)
I	1746.25	145.49	17.83	1.42
II	1669.29	86.37	16.37	0.84
III	1635	15	16.13	0.05

## Data Availability

Data are contained within the article.
